# Enzyme-Assisted Tenderization and Vitamin E-Loaded Liposome Coating for Garlic Scape Quality Enhancement

**DOI:** 10.3390/foods15010008

**Published:** 2025-12-19

**Authors:** Juhyun Kim, Jiseon Lee

**Affiliations:** 1 Department of Food Science and Biotechnology of Animal Resources, Konkuk University, Seoul 05029, Republic of Korea; kimjh25@konkuk.ac.kr; 2 School of Animal, Food Science and Marketing, Konkuk University, Seoul 05029, Republic of Korea

**Keywords:** enzyme treatment, garlic scapes, liposome, nutrition, tenderness

## Abstract

Older adults and patients with masticatory and deglutition disorders often experience difficulties consuming tough, fibrous vegetables. The enzymatic and liposomal conditions for softening garlic scapes were optimized while simultaneously enhancing their nutritional value through vitamin E fortification. Enzymes (Plantase UF and Plantase PT) were applied at varying concentrations and incubation times to determine optimal tenderization conditions, followed by the application of vitamin E-loaded liposomes. The physicochemical, microstructural, and color characteristics of the scapes and liposomal systems were evaluated. Enzymatic treatment significantly (*p* < 0.05) decreased hardness and increased adhesiveness, indicating effective cell wall disruption. Plantase PT hydrolyzes pectin in the middle lamella, promoting cell separation and softening, and maintains higher activity than Plantase UF, confirming its suitability for the consistent tenderization of fibrous vegetables. Its stability ensures reliable and uniform softening for real-world fibrous vegetable processing. Enzyme–vitamin E co-encapsulation balanced texture and nutrition by enlarging particles and lowering the ζ-potential (*p* < 0.05). Liposomal encapsulation preserved enzyme activity during processing and enabled sustained vitamin E delivery to scape tissues. Compared with untreated control, vitamin E liposomes provided controlled softening and improved nutrient stability. This highlights the potential of enzyme–liposome systems in developing tenderized older adult-friendly diets using fibrous plants.

## 1. Introduction

The global population is aging rapidly, and the proportion of individuals over 65 years of age is expected to double by 2050 [1]. Older adults, particularly those over 75 years, are vulnerable to malnutrition owing to reduced food intake, limited dietary diversity, and impaired mastication, which are closely linked to frailty, weakened immunity, and the avoidance of fibrous vegetables [2,3].

The garlic scape (*Allium sativum* L.) is a flowering stalk that develops during garlic cultivation. It is commonly removed after harvest and is often used as an organic fertilizer in the field [4]. Garlic scapes are also valued for their bioactive compounds, including flavonoids and polyphenols, which exhibit antioxidant activities and contribute to lipid metabolism [5,6]. Vegetables of the *Allium* genus, such as onions, leeks, and scallions, are known for the fibrous tissues in their leaf sheaths and pseudo-stems, where the accumulation of cellulose, hemicellulose, and lignin contributes to their tough texture [7,8,9]. The fibrous characteristics of garlic scapes make them difficult to chew, particularly for older adults, highlighting the need for texture modification strategies [10]. Hence, tenderization methods should be explored to enhance their suitability in the diets of older adults. Despite their nutritional value, the intake of tough plant tissues is often limited among this population. Conventional methods such as boiling, grinding, or pureeing can soften their texture, but they usually result in nutrient loss or undesirable changes in appearance. Enzymatic treatments provide an alternative by selectively degrading structural polysaccharides such as cellulose, hemicellulose, and pectin. Cellulase catalyzes the hydrolysis of β-1, 4 glycosidic bonds in cellulose, releasing glucose monomers and thereby weakening the rigidity of the cell wall. Hemicellulases degrade hemicellulose into oligosaccharides and monosaccharides, contributing to the loosening of the cell wall matrix. Pectinases hydrolyze the α-1,4-glucosidic bonds in pectin, a major component responsible for intercellular adhesion within plant tissues [11]. Such disruption of intercellular pectin enhances the accessibility of cellulose and hemicellulose, thereby contributing to efficient softening during enzymatic treatment [12]. In particular, cellulases and pectinases effectively tenderize vegetables while retaining their natural appearance and sensory attributes [13]. This strategy is recognized as a promising approach for developing texture-modified vegetables that are both palatable and nutritionally beneficial for older adults. Such enzymatic modifications contribute to the softening of plant tissues while maintaining the overall tissue structure, thereby achieving a texture suitable for senior citizens.

Encapsulation systems, such as emulsions and liposomes, have been employed to enhance the stability and control the release of sensitive nutrients, while maintaining soft and safe textures suitable for individuals with mastication or swallowing difficulties. Emulsion–gel systems have been developed to deliver lipophilic bioactive compounds and improve the rheological properties of dysphagia-friendly foods [14]. Liposome technology has been applied to obtain higher bioavailability of vitamin C for use in the production of dietary supplements, nutraceuticals, and functional foods for seniors [15]. Although emulsion systems are beneficial, they frequently encounter issues, such as creaming, coalescence, and textural limitations, which can complicate the development of dysphagia-friendly foods. In contrast, liposomes, characterized by a phospholipid bilayer that encapsulates both hydrophilic and lipophilic nutrients, provide enhanced protection and controlled release under various conditions, including processing, storage, and gastrointestinal conditions [16]. Specifically, for lipophilic compounds such as vitamin E, liposomal encapsulation has demonstrated greater oxidative stability and bioavailability than conventional emulsion-based or protein-based systems, owing to the protective phospholipid bilayer that enables gradual release during digestion [17,18].

Vitamin E (α-tocopherol) is a lipophilic antioxidant that has been reported to prevent oxidative stress, delay aging, and reduce the risk of cardiovascular disorders by modulating lipid metabolism [19]. Additionally, a correlation exists between elevated dietary vitamin E consumption and reduced cognitive deterioration rates in older adults [20], suggesting the potential role of vitamin E in preserving cognitive function during aging [21]. However, the poor water solubility and high susceptibility to oxidative degradation of this vitamin limits its direct incorporation into food matrices [16]. Liposome-based encapsulation has been widely utilized to enhance the stability and bioavailability of bioactive compounds, including vitamins and antioxidants, and has been successfully applied to food systems for nutrient fortification [22,23].

Nutritional studies have indicated that appropriate dietary interventions can delay the onset of age-related disorders and support cognitive health [2,10]. In this context, vitamin E-enriched tenderized garlic scapes may serve as a senior-friendly functional food that combines improved palatability with potential cardiovascular and cognitive health benefits through sustained antioxidant action. Enhancing the palatability and accessibility of garlic scapes through tenderization could increase their utilization in older adult–friendly diets, while also contributing to balanced nutrition.

The objective of this study was to develop a dual strategy for garlic scapes by combining dietary fiber-degrading enzymes with vitamin E-loaded liposomes. This approach aimed at achieving simultaneous tenderization and fortification, thereby enhancing the functional and nutritional value of garlic scapes for application in older adult-friendly foods.

## 2. Materials and Methods

### 2.1. Materials

Garlic scapes were purchased from a local market (Seoul, Republic of Korea). Plantase UF and Plantase PT were provided by Bision Corporation (Seoul, Republic of Korea). Both enzymes originate from *Aspergillus niger*; Plantase PT is a pectinase-dominant preparation, while Plantase UF is a multi-enzyme blend containing pectinase, β-glucanase and arabinase activities. Water-soluble phosphatidylcholine (Lipoid S75; Lipoid GmbH, Ludwigshafen, Germany) was used to prepare the liposomes. These preparations were selected because they present two enzyme systems with distinct substrate specificities, allowing comparisons between moderate and extensive tenderization effects in fibrous plant tissues. Plantase UF contains pectinase together with β-glucanase and arabinase, producing controlled softening, whereas Plantase PT is rich in pectinase and induces more extensive degradation of the pectic middle lamella [24]. Vitamin E (DL-α-tocopherol) was purchased from TCI (Tokyo, Japan). Chemicals used for enzymatic activity analysis included polygalacturonic acid solution (Sigma-Aldrich, Burlington, MA, USA), iodine (FUJIFILM Wako Pure Chemical Corporation, Osaka, Japan), potassium iodine (Daejung Chemical and Metals, Siheung-si, Gyeonggi-do, Republic of Korea), sodium carbonate (Samchun Chemical, Seoul, Republic of Korea), sulfuric acid (90%; Daejung Chemical and Metals), potato starch (Daejung Chemical and Metals), and 0.1 M sodium thiosulfate (Yakuri Pure Chemical, Kyoto, Japan). All other reagents were of analytical grade or higher.

### 2.2. Optimization of Enzyme Concentration and Treatment Time

#### 2.2.1. Sample Preparation for Enzyme Optimization

Various conditions were tested to optimize the enzyme concentration. The garlic scapes were cut into 4 cm lengths and immersed in 40 mL of 1–3% enzyme solution for 24 h. To optimize the treatment time, a 2% enzyme solution was prepared, and the garlic scapes were immersed for 12, 24, and 48 h. These samples were subsequently used for color, texture, and microscopic analyses. The appearance of the garlic scape samples was observed using a camera (EOS 100D; Canon, Tokyo, Japan).

#### 2.2.2. Color Measurement

The color values for each of the different garlic scapes were measured with a colorimeter (CR-400; Konica Minolta Sensing Inc., Tokyo, Japan) calibrated using a white standard plate (L* = 96.06; a* = −0.38; b* = 1.23). The instrument was placed on different sections of the garlic scape surface during analysis, and measurements were taken at three randomly selected points along each sample. To compare the visual color differences among the samples, the total color difference (*Δ*E) was calculated using the following equation:

Total color difference (*Δ*E) = √[(*Δ*L*)^2^ + (*Δ*a*)^2^ + (*Δ*b*)^2^] (1)

Color quality evaluation based on L*, a*, and b* parameters is widely applied for assessing appearance changes in vegetables during processing [25].

#### 2.2.3. Texture Profile Analysis Method

The measured textural properties included hardness and adhesiveness. Texture profile analysis of the garlic scape samples was performed using a CT3-1000 texture analyzer (Brookfield Engineering Laboratories, Stoughton, MA, USA) equipped with a TA22 probe. The selected settings were a 900 g trigger load and 2.5 mm/s test speed for the compression test type. Samples were compressed in the transverse direction (Figure S1).

Hardness was defined as the peak force recorded during the first compression cycle, representing the force required to achieve a given deformation. Adhesiveness was determined as the negative area under the force–time curve during the first compression.

#### 2.2.4. Optical Microscopic Analysis

Garlic scape samples were observed under an optical microscope (Olympus CX31; Olympus Optical Co., Ltd., Tokyo, Japan), and images were captured using a charged-coupled device (CCD) camera (3.0 M, Olympus Optical Co., Ltd. Tokyo, Japan) at ×400 magnification. Thin hand-cut sections were placed on glass slides and observed without staining.

#### 2.2.5. Enzyme Activity Assay

For enzymatic activity analysis, a pectinase enzymatic assay (Sigma-Aldrich, Burlington, MA, USA) was used. A 0.5% (*w*/*v*) polygalacturonic acid solution (Sigma-Aldrich) was prepared and adjusted to pH 4.0 using 1 N sodium hydroxide. A 50 mM iodine/200 mM potassium iodide solution was prepared using iodine (FUJIFILM Wako Pure Chemical Corporation, Osaka, Japan) and potassium iodine (Daejung Chemical and Metals, Gyeonggi-do, Republic of Korea). A 1 M sodium carbonate solution was prepared from sodium carbonate (Samchun Chemical, Seoul, Republic of Korea), and 2 N sulfuric acid was obtained by diluting concentrated sulfuric acid (90%; Daejung Chemical and Metals). Additionally, a 1% (*w*/*v*) starch solution, prepared from potato starch (Daejung Chemical and Metals), served as an indicator.

The blank consisted of polygalacturonic acid solution without enzyme, processed identically to the samples. This assay is based on the iodine−thiosulfate titration principle, in which the hydrolysis of polygalacturonic acid reduces iodine binding; therefore, a lower titration volume corresponds to higher enzymatic activity.

For the enzyme assay, 4.9 mL of polygalacturonic acid solution and 0.1 mL of sample solution were mixed in a 50 mL conical tube and incubated at 25 °C with shaking for 5 min. The enzyme samples were used without dilution unless otherwise stated. Subsequently, 5 mL of iodine/potassium iodide solution and 1 mL of sodium carbonate solution were added, and the mixture was kept in the dark for 20 min. The enzymatic reaction was terminated by adding 2 mL of 2 N sulfuric acid with gentle mixing, followed by one drop of 1% starch solution as an indicator. The mixture was titrated with 0.1 M sodium thiosulfate (Yakuri Pure Chemical, Kyoto, Japan) until the solution became colorless, and the titration volume was recorded. Enzyme activity was calculated using the following equation:

Enzyme activity (units/mL) = [(A − B) × N × 1000]/(t × V_e_) (2)

where A is the titration volume (mL) of the blank sample, B is the titration volume (mL) of the enzyme-treated sample, and N, t, and V_e_ represent the normality of sodium thiosulfate, reaction time, and enzyme volume, respectively. The reaction time (t) used for activity calculation was 5 min. One unit (U) of pectinase activity was defined as the amount of enzyme releasing 1 µmol of galacturonic acid per hour at pH 4.0 and 25 °C, according to the Sigma-Aldrich protocol. All measurements were performed in triplicate.

### 2.3. Optimization Strategy of Vitamin E Content

To optimize the vitamin E (DL-α-tocopherol; TCI, Tokyo, Japan) concentration, lecithin was dispersed in distilled water to obtain a final concentration of 1% (*w*/*v*). Vitamin E was directly added to the lecithin dispersions and homogenized at different concentrations (0, 0.025, 0.05, 0.1, and 0.2% *w*/*v*). The volume of the mixture was adjusted, and the mixture was stirred at 700 rpm for 30 min. The resulting dispersions were homogenized at 10,000 rpm for 3 min using a high-speed homogenizer, followed by sonication at 60 W for 3 min using an ultrasonicator (Model HD2200; Bandelin Electronic GmbH & Co., KG, Berlin, Germany) to produce vitamin E-loaded liposomes. The particle size, polydispersity index, and ζ-potential were measured by dynamic light scattering with a Zetasizer ZS 90 instrument (Malvern Instruments, Worcestershire, UK). The samples were diluted 100 times with distilled water. The prepared liposomes were evaluated and compared with a 2% (*w*/*v*) Plantase PT solution and liposomes coated with 2% (*w*/*v*) Plantase PT. Measurements were performed in triplicate at 25 °C, and the results were expressed as mean ± standard deviation.

### 2.4. Characterization of Liposome-Loaded Garlic Scapes

#### 2.4.1. Preparation of Liposomes

To prepare vitamin E-loaded liposomes, 1% (*w*/*v*) lecithin and 0.1% (*w*/*v*) vitamin E were dispersed in distilled water. The mixture was homogenized using a high-speed homogenizer at 10,000 rpm for 3 min, and then sonicated using an ultrasonicator at 60 W for 3 min. To prepare the enzyme–liposome mixture, liposomes were mixed with a 2% (*w*/*v*) enzyme solution at 400 rpm for 5 min.

#### 2.4.2. Liposome Treatment of Garlic Scapes

Garlic scapes were cut into 4 cm lengths and immersed in 40 mL distilled water (blank sample; C), blank liposome (B), 2% enzyme solution (E), enzyme combined with blank liposome (BE), vitamin E-loaded liposome (L), or enzyme combined with vitamin E-loaded liposome (LE) for 3, 6, and 12 h under 4 °C refrigeration. The appearance of the garlic scape samples was observed using a camera (EOS 100D; Canon, Tokyo, Japan).

#### 2.4.3. Color Profile Analysis

The color of the liposome-loaded garlic scape samples was evaluated using a colorimeter under the same conditions as described in Section 2.2.2. Color quality assessment based on L, a*, and b* values is commonly used to monitor appearance changes in plant-based foods [25].

#### 2.4.4. Texture Profile Analysis

The textural properties of the liposome-loaded garlic scape samples were evaluated using a CT3-1000 texture analyzer under the same conditions described in Section 2.2.3.

#### 2.4.5. Optical Microscopic Images

Liposome-loaded garlic scape samples were observed under an optical microscope, and images were obtained using a CCD camera at ×400 magnification.

#### 2.4.6. Enzyme Activity

The enzyme activity of the samples was evaluated enzymatically using pectinase under the conditions described in Section 2.2.5.

#### 2.4.7. Vitamin Content Analysis

To determine whether vitamin E was absorbed and enriched in the vegetables after the application of vitamin E-loaded liposomes, the vitamin E content of the treated vegetables was quantified using high-performance liquid chromatography (HPLC), following the method described in [26] with some modifications. After 3 h of storage, approximately 1 g of tissues was collected from each treatment. Only the vitamin E-loaded liposome (L) and enzyme combined with the vitamin E-loaded liposome (LE) groups were analyzed for vitamin E content. The surface moisture was blotted, and 15 mL of acetone (HPLC grade) was added. The samples were then homogenized at 25 °C for 1 min. The extraction was repeated twice, and the combined acetone extracts were used for analysis. The 2 mL aliquot of the acetone extract was withdrawn, filtered through a 0.45 µm nylon syringe filter (Whatman International Ltd., Maidstone, UK), transferred to an HPLC vial, and analyzed.

HPLC analyses were performed using the Agilent 1100 system (Agilent Technologies, Santa Clara, CA, USA) equipped with a diode array detector set at 295 nm. Separation was achieved on a YMC C-30 column (250 × 4.6 mm, 5 µm; YMC Co., Ltd., Kyoto, Japan) at 20 °C with a flow rate of 1.0 mL/min. The mobile phases were (A) methanol/water (95:5, *v*/*v*) containing 5 mM ammonium formate, and (B) tertbutyl methyl ether/methanol/water (90:7:3, *v*/*v*/*v*). The gradient program ranged from 0% to 100% B over 45 min. The injection volume was 20 µL.

Quantification was performed by external calibration using DL-α-tocopherol (the same standard used in liposome preparation). Peak identity was confirmed by the retention time and ultraviolet spectra at 295 nm, and concentrations were calculated from the calibration curve and normalized to the fresh weight of the sample.

### 2.5. Statistical Analysis

All measurements were performed on at least three sample preparations and expressed as the mean ± standard deviation. Statistical data were compared using Duncan’s multiple range test (*p* < 0.05) and one-way analysis of variance in SPSS statistical software (version 24.0; SPSS Inc., Chicago, IL, USA).

## 3. Results and Discussion

### 3.1. Color Characteristics of Enzyme-Treated Garlic Scapes

The color parameters of the garlic scapes subjected to Plantase UF and Plantase PT treatments are presented in Table 1. The lightness (L*), yellowness (b*), and redness (a*) of the enzyme-treated samples increased compared to those of the untreated samples. At 1% and 3% concentrations of Plantase UF, the L* and *Δ*E values were significantly higher than those of the control (*p* < 0.05); however, no significant difference was observed at 2% (*p* > 0.05). Similar trends were observed for Plantase PT. The *Δ*E values of Plantase UF were consistently higher than those of Plantase PT, indicating a more superficial and partial degradation of cell wall polysaccharides by UF, which likely enhances surface reflectance without significant tissue breakdown. In contrast, the hydrolytic activity of PT presumably induced pigment effusion and moisture-related scattering, leading to less uniform *Δ*E patterns despite more extensive structural alteration.

Previous studies have shown that enzyme treatment can alter the cell wall composition, thereby influencing pigment stability [27]. The observed increases in L*, a*, and b* values after enzymatic treatment suggest that fiber-degrading enzymes modify the surface structure and pigment stability of garlic scapes. Similar brightening effects have been reported in enzyme-treated vegetables, in which the partial hydrolysis of cell wall polysaccharides led to increased light scattering and enhanced reflectance [28]. Moreover, ΔE, which quantifies the color difference between the treatment and control, serves as an indicator of the visual changes caused by physicochemical alterations. According to established thresholds, a ΔE value between 3 and 6 indicates a noticeable difference, while values from 6 to 12 suggest a distinct color variation, and values exceeding 12 represent markedly different color tones [29].

**Figure 1:**
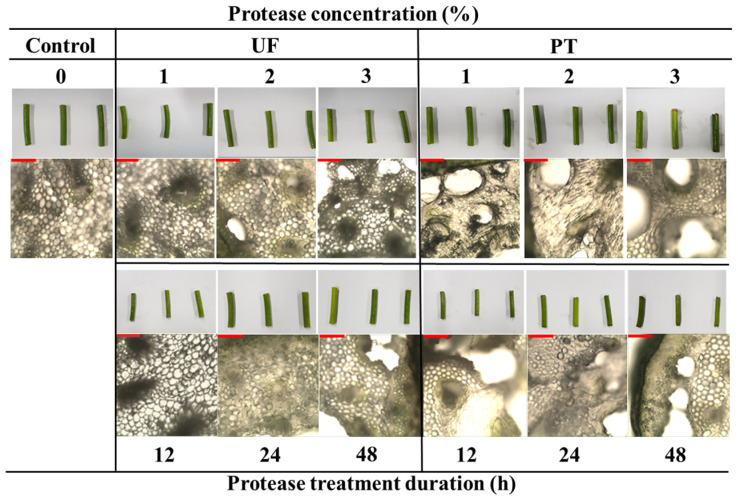
Appearance and optical microscopy images of garlic scapes treated with different Plantase types, concentrations, and treatment durations. Control, untreated enzyme. UF and PT refer to Plantase UF and Plantase PT, respectively. Scale bar is 10 μm.

The duration of the enzymatic process plays a crucial role in determining textural properties [30]. The time-dependent changes indicated that the L* and b* values of the Plantase UF group increased after 12 h, whereas those of the Plantase PT group demonstrated less consistent effects. These results confirm that enzymatic degradation of structural polysaccharides modifies the optical properties of tissues, which is consistent with previous findings that cellulase and pectinase can alter chromatic stability by affecting the tissue microstructure [13]. Representative images (Figure 1) visually corroborate the lighter appearance and disrupted cell integrity following enzyme treatment.

### 3.2. Texture Profile of Enzyme-Treated Samples

Texture analysis (Table 2) demonstrated that the enzymatic treatments significantly affected the hardness and adhesiveness of the samples. In the Plantase UF group, no statistically significant differences were observed in hardness or adhesiveness compared with the control. The Plantase UF-treated samples initially showed a temporary increase in hardness at lower concentrations followed by a decrease at higher concentrations or extended treatment durations after 12 h of treatment. This transient behavior was likely caused by the mixed composition of Plantase UF, which contains β-glucanase and arabinase in addition to pectinase. Because these enzymes mainly target hemicellulose side chains rather than the pectin backbone, their contribution to structural softening is inherently limited.

In contrast, Plantase PT exhibited a significant concentration-dependent reduction in hardness, along with a significant increase in adhesiveness as the enzyme concentration increased. These results indicate that Plantase PT had a more pronounced softening effect on garlic scapes than Plantase UF. Furthermore, at equivalent concentrations, the hardness values of the Plantase PT-treated samples were significantly lower than those of the Plantase UF-treated samples. In contrast, the adhesiveness was significantly higher in the Plantase PT group. This suggests that Plantase PT is more effective than Plantase UF at promoting tissue softening and modifying the textural properties of garlic scapes. The observed reduction in hardness following enzymatic treatment was attributed to the partial hydrolysis of cell wall polysaccharides, including cellulose, hemicellulose, and pectin [11].

The Plantase UF-treated samples maintained moderate hardness reduction and low adhesiveness, suggesting that Plantase UF provided a controlled tenderization effect. In contrast, Plantase PT treatment caused a drastic reduction in hardness after 48 h, accompanied by a sharp increase in adhesiveness, indicating excessive tissue breakdown and water absorption. These findings are related to enzyme-specific activity, with Plantase UF producing stable softening and Plantase PT causing over-softening. This highlights the importance of enzyme selection in modifying the texture of fibrous vegetables. The time-dependent decrease in hardness and increase in adhesiveness indicated the progressive hydrolysis of structural polysaccharides within the cell walls of garlic scapes. Prolonged exposure to the enzyme allows deep diffusion and more complete cleavage of cellulose, hemicellulose, and pectin linkages, leading to the weakening of the middle lamella and the collapse of the parenchymal tissues. Similar time-related softening behavior has been observed in other vegetable matrices, such as carrots and asparagus, where extended enzymatic contact promotes cell separation and turgor loss [31,32].

The pronounced tenderization effect of Plantase PT can be primarily attributed to its higher pectinolytic activity. Previous studies have demonstrated that pectin-degrading enzymes, such as polygalacturonase and pectate lyase, directly hydrolyze the pectic substances of the middle lamella, which are responsible for intercellular adhesion and tissue rigidity [24]. Pectin degradation results in cell separation and tissue softening, which is consistent with previous findings that pectin and hemicellulose structure strongly influence plant texture and that pectin hydrolysis reduces cell wall integrity and water-binding capacity [33,34]. These enzymatic reactions lead to distinct structural modifications in the cell wall structure. Specifically, Plantase PT promotes the breakdown of the pectic network in the middle lamella, resulting in cell detachment and increased adhesiveness, whereas Plantase UF primarily loosens the cellulose–hemicellulose matrix, producing moderate softening while maintaining cell adhesion. In contrast, enzymes targeting hemicellulose or side-chain components, such as β-glucanase and arabinase, play a supplementary role by enhancing porosity and enzyme penetration but exhibit a limited capacity to induce softening independently [35]. This mechanistic distinction explains why the Plantase UF-treated samples, which contained mixed β-glucanase and arabinase activities, showed only moderate textural changes. In contrast, PT-treated samples displayed substantial softening and increased adhesiveness.

### 3.3. Microstructural Alterations Induced by Enzymes

Optical microscopy (Figure 1) further revealed differences between the enzyme treatments. The control samples retained compact and intact structures, whereas the enzyme-treated samples exhibited loosening of the cell walls and pore formation. Plantase UF treatment caused only a slight loosening of the tissue structure without apparent cell wall rupture, indicating limited enzymatic penetration and mild hydrolytic activity. The modest penetration depth is consistent with the low pectin-targeting capability of Plantase UF, which preserves much of the structural backbone. In contrast, Plantase PT induced severe collapse and enlarged pores, in line with a greater reduction in hardness and higher adhesiveness. These microstructural findings confirm that the type of enzyme and treatment time dictate the extent of tissue disintegration and the resulting textural outcomes. This is consistent with studies demonstrating that the enzymatic hydrolysis of pectin, a major component of the plant cell wall, leads to cell turgor loss and compaction, thereby altering the textural properties of the material [35].

### 3.4. Residual Enzyme Activities of Treated Solution

**Figure 2:**
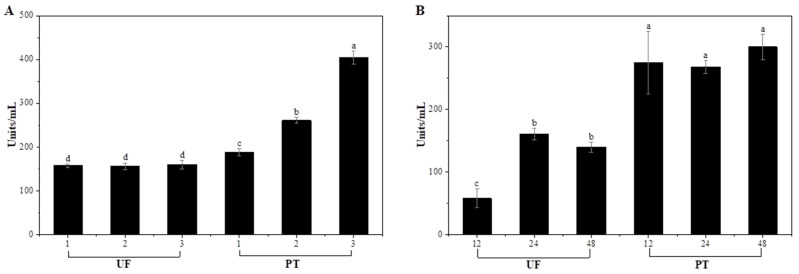
Enzyme activity (units/mL) of garlic scapes treated with different Plantase types, concentrations (**A**), and treatment durations (**B**). UF and PT refer to Plantase UF and Plantase PT, respectively. ^a–d^ Values with different superscript letters are significantly different (*p* < 0.05).

Enzymatic activity was determined based on the amount of galacturonic acid residues released from polygalacturonic acid, which reflects the hydrolysis of α-1,4-glycosidic linkages catalyzed by pectinase. Through hydrolysis of α-1,4-glycosidic linkages between galacturonic acid residues, pectinase breaks down polygalacturonic acid into smaller oligomers and monomeric galacturonic acid. The resulting increase in galacturonic acid equivalents thus represents the depolymerization activity of the enzyme [36]. The results for each treatment time are presented in Figure 2. The activity of Plantase UF showed no statistically significant differences across the range of concentrations employed, implying that its enzymatic function was not concentration-dependent under these specific conditions (*p* > 0.05). This result may be attributed to substrate saturation or limited enzyme accessibility to the cell wall components, which restricted further increases in the catalytic rate despite higher enzyme concentrations. The lowest activity of Plantase UF was observed after 12 h of incubation (*p* < 0.05). In contrast, the highest activity was recorded at 24 h, followed by a significant decrease at 48 h. This fluctuation suggests that prolonged incubation may lead to partial enzyme inactivation, possibly because of conformational instability or substrate depletion. All treatment groups exhibited higher Plantase PT enzymatic activity than Plantase UF activity, and no significant differences were observed among the incubation times. This consistent activity profile indicates that Plantase PT retains its catalytic efficiency regardless of the treatment duration or vegetable matrix, suggesting greater stability under the experimental conditions. The stable performance of Plantase PT aligns with its observed softening efficiency and the uniform textural modification of garlic scapes. In this study, enzymatic activity was evaluated based on pectinase-catalyzed hydrolysis of polygalacturonic acid, as pectin degradation is a primary determinant of tissue softening in plant materials. Nevertheless, further assessment of other enzymatic components, such as β-glucanase and arabinase in Plantase UF, would provide additional insight into their respective contributions to the overall tenderization process. Previous studies have reported that the intrinsic structural features of enzymes enable them to maintain their catalytic activity over prolonged processing periods under various environmental stresses [37,38]. Stability is a critical factor in industrial applications to ensure predictable and consistent product quality [39]. Together with the color, appearance, and texture results, these findings support the selection of 2% Plantase PT as the optimal enzyme concentration, as it provides effective tenderization without causing excessive discoloration or structural collapse.

### 3.5. Optimization of Vitamin E Content

Liposomes were designed as encapsulation carriers to enhance vitamin E delivery for nutrition in the older adult population. For successful development of vitamin E-loaded liposomes, the vesicle structure must remain stable even after vitamin E incorporation.

Therefore, determining the appropriate concentration of vitamin E is critical for maintaining liposomal integrity. Various concentrations of vitamin E were applied, and the stability of the resulting liposomes was evaluated before determining their suitability for enzyme encapsulation. The liposomal formulations with different vitamin E concentrations exhibited distinct physicochemical properties (Table 3). At low vitamin E concentrations (0.025–0.05%), particle size exceeded 1 µm with a low ζ-potential, indicating poor colloidal stability. In contrast, at 0.1% vitamin E, the particle size stabilized at approximately 291 nm with a high ζ-potential. As colloidal systems with an absolute ζ-potential above 30 mV are generally considered electrostatically stable, the value observed at 0.1% vitamin E indicates a stable liposomal dispersion with improved vesicle integrity [40]. At 0.2% vitamin E, however, the particle size increased to 404 nm, and the ζ-potential dropped to 13.73 mV, demonstrating a loss of stability at higher vitamin E loading. This suggests that excessive vitamin E disrupts the phospholipid bilayer packing, leading to vesicle swelling and reduced electrostatic repulsion. Therefore, 0.1% vitamin E represent the optical concentration that enhances bilayer compactness and surface charge density without causing destabilization.

The incorporation of the enzyme (LE) resulted in larger particles with a reduced ζ-potential, reflecting partial bilayer disruption and decreased electrostatic repulsion. These results are consistent with previous reports showing that the inclusion of lipophilic bioactive compounds can modify phospholipid packing and surface charge characteristics [16].

### 3.6. Color and Microscopic Observation

This section presents the color profile analysis results for the garlic scapes treated with the different liposomal systems (Table 4). Compared to the control group (C), the treatments using B and L exhibited minor increases in brightness (L*) and yellowness (b*); however, the overall total color difference (*Δ*E) remained low (<5), indicating minimal visual changes. This suggests that liposome immersion alone did not cause prominent discoloration or structural alteration of the garlic scape tissues.

Among the enzyme-treated groups, E and BE induced greater color changes, reflecting noticeable tissue breakdown and increased light scattering. This indicates that enzymatic treatment can alter the structural integrity of plant tissues, consequently affecting their optical properties [41]. LE exhibited the lowest *Δ*E among the enzyme-loaded treatments, suggesting that the co-loading of vitamin E helped mitigate tissue degradation and oxidative pigment loss. This may be attributed to the antioxidant capacity of vitamin E, which can stabilize pigments and limit enzymatic over-degradation. This interpretation is consistent with previous findings that antioxidant compounds help maintain the color stability of plant-derived materials by suppressing enzymatic browning and protecting the pigments from oxidative deterioration [42,43].

**Figure 3:**
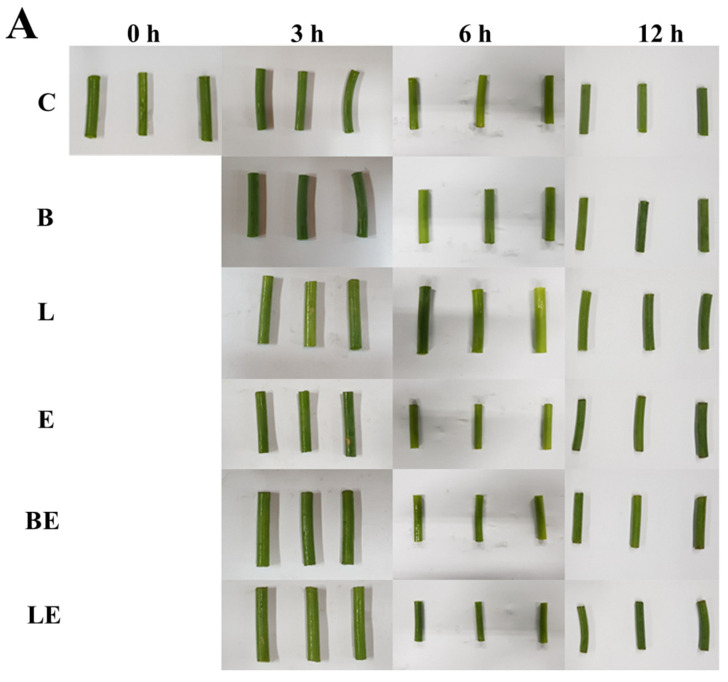

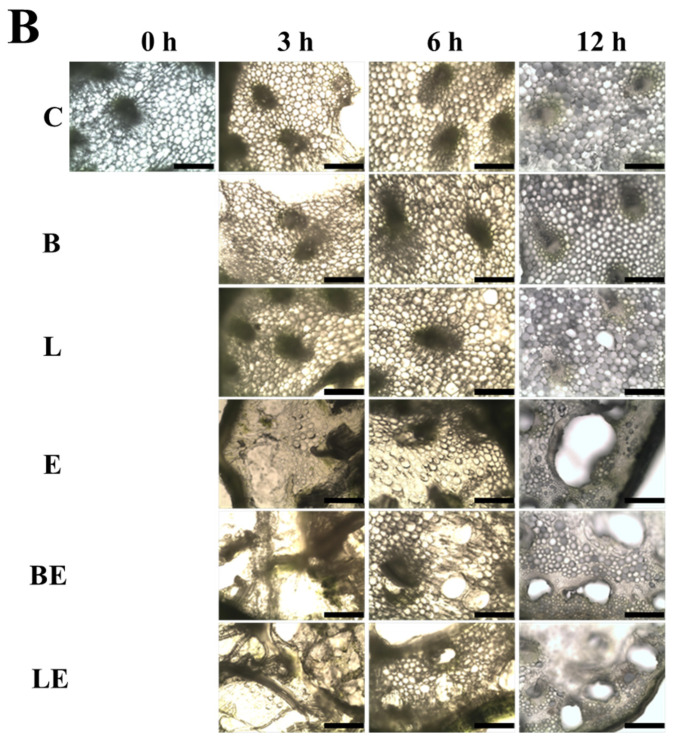
Appearance (**A**) and optical microscopy images (**B**) of garlic scapes treated with different liposome formulations: C, untreated sample; B, blank liposome; L, vitamin E-loaded liposome; E, 2% enzyme solution; BE, enzyme combined with blank liposome; LE, enzyme combined with vitamin E-loaded liposome. Scale bar is 10 μm.

Microscopic observations (Figure 3) further support these findings. The non-enzymatic groups (B and L) exhibited a compact and well-preserved cellular architecture without discernible deformation or pore formation, indicating that immersion in the liposomal solution alone did not alter the tissue integrity of the garlic scapes. In contrast, the enzyme-treated samples (E, BE, and LE) exhibited disrupted tissue structures and enlarged pores, confirming that enzymatic hydrolysis induced the morphological degradation of the garlic scape tissues. The LE group maintained a more cohesive morphology with partial pore formation, supporting the hypothesis that vitamin E provides structural protection by suppressing excessive oxidation and enzymatic degradation. The microscopic observations were consistent with the colorimetric data, suggesting that antioxidant activity helps preserve tissue integrity and stabilizes the overall color appearance.

### 3.7. Texture

The texture profiles (hardness and adhesiveness) of the garlic scapes treated with C, B, L, E, BE, and LE are presented in Table 5. Compared with the control (C), all the enzyme-containing groups (E, BE, and LE) showed reduced hardness after 6 h, indicating enzymatic softening. Among them, BE showed the most pronounced decrease (5690 ± 781 g). The tenderization effect of Plantase PT is mainly attributed to its high pectinolytic activity, which directly degrades pectin substances in the middle lamella responsible for cell-to-cell adhesion. The breakdown of these pectins weakens intercellular connections, leading to cell separation, increased adhesiveness, and a marked reduction in tissue hardness. In comparison, LE exhibited moderate reduction (6323 ± 874 g), suggesting that vitamin E co-loading may have partially hindered enzyme diffusion or slowed degradation. This moderate softening is desirable in texture-modified foods for older adults, as excessive collapse can impair palatability and structural integrity.

Treatment with L alone did not significantly change the hardness compared to treatment with C, confirming that vitamin E itself does not affect tissue texture. Similarly, B did not cause a meaningful reduction, reinforcing the conclusion that enzymatic activity is the primary contributor to tenderization. This interpretation is consistent with those of previous studies reporting that modifications in pectin structure and solubilization govern the mechanical behavior of plant tissues, whereas the overall texture remains unaffected in the absence of targeted polysaccharide degradation [43,44].

Adhesiveness values ranged from 1.80 to 3.33 mJ and were not significantly affected by any treatment, indicating that the liposome-based systems did not compromise the stickiness or handling properties of the scapes. This stability in adhesiveness, even with enzymatic softening, indicates that the overall pectic network, which is vital for cell-to-cell adhesion, was not excessively degraded in a manner that would lead to undesirable stickiness [45].

Microscopic analysis (Figure 3B) revealed that the LE-treated tissues displayed partial pore formation while maintaining structural regions, unlike the E and BE tissues, which showed more extensive breakdown. This is visual evidence of the balanced tenderization effect achieved by co-loading vitamin E. This confirms that LE treatment achieved a controlled tenderization effect by balancing enzymatic softening with antioxidant-mediated tissue protection.

These results suggest that LE provides a balanced tenderization effect—providing sufficient softening for ease of mastication without causing excessive tissue collapse—making it ideal for older adult-friendly, texture-modified vegetables.

### 3.8. Enzyme Activity and Stability in Liposomal Formulations

**Figure 4:**
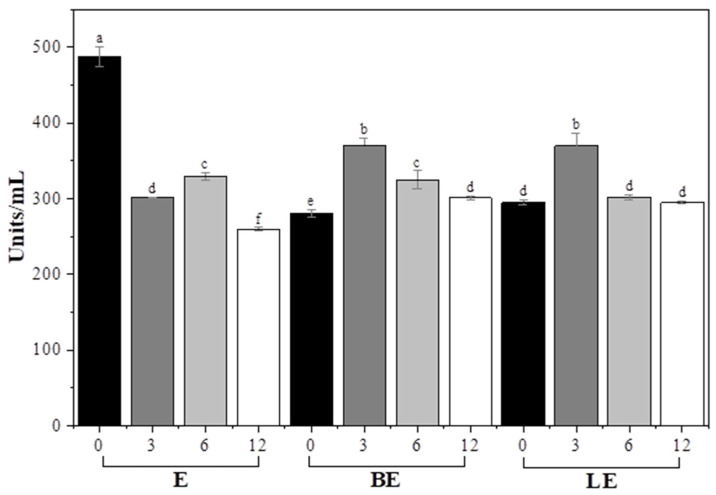
Enzyme activity (units/mL) of garlic scapes treated with different liposome formulations for various durations (0, 3, 6, and 12 h). E, 2% enzyme solution; BE, enzyme combined with blank liposome; LE, enzyme combined with vitamin E-loaded liposome. ^a–f^ Values with different superscript letters are significantly different (*p* < 0.05).

The enzyme activity patterns and colloidal stability of the liposomal systems used in the garlic scape treatments were evaluated. Enzyme activity (Figure 4) was the highest in group E at the earliest point (3 h), but declined rapidly over time, likely due to enzymatic denaturation or inactivation in the external aqueous environment.

By contrast, BE and LE exhibited lower initial activity but maintained stable enzymatic activity throughout the 12 h incubation period. This stability suggests that liposomal encapsulation can protect the enzyme from environmental degradation, possibly by limiting its direct exposure to inhibitors or proteases in the medium [46].

Physicochemical measurements of the treatment media (Table 6) revealed that B and L maintained relatively consistent particle sizes and ζ-potential, indicating stable colloidal behavior. However, in BE and LE, the particle size increased significantly, whereas the ζ-potential decreased (e.g., down to ~10 mV), indicating destabilization caused by enzyme–lipid interactions. Comparable trends have been reported in previous studies investigating enzyme interaction with phospholipid vesicles. Interaction between phospholipase C and DPPC and DOPC liposomes resulted in a pronounced increase in particle size and a reduction in ζ-potential, which was attributed to enzyme absorption and the partial hydrolysis of the lipid bilayer that weakened electrostatic stabilization [47]. This mechanism is consistent with the present results, where the addition of the enzyme in the BE formulation led to a sharp reduction in ζ-potential and an increase in particle size. In BE, the ζ-potential neared 0 mV at 6 h, suggesting pronounced aggregation. The enzyme likely interacted with the lipid surface through hydrophobic and electrostatic domains, partially neutralizing surface charges and disturbing the hydration layer, which facilitated vesicle–vesicle bridging and coalescence. Nevertheless, enzymatic activity was preserved in LE, implying that vitamin E conferred protective effects that maintained catalytic function despite colloidal destabilization. The incorporation of vitamin E likely contributed to the preservation of enzymatic activity by reinforcing the lipid bilayer and reducing oxidative degradation, which helped maintain a stable microenvironment during processing [18]. These differences demonstrate that while enzyme addition destabilized blank liposomes by disrupting surface charge and bilayer structure, vitamin E incorporation counteracted this effect.

LE demonstrated the most favorable balance between enzyme stability and delivery performance, highlighting its utility as a protective and sustained-release system for the enzymatic processing of fibrous vegetables.

### 3.9. Vitamin E Enrichment of Garlic Scapes

**Figure 5:**
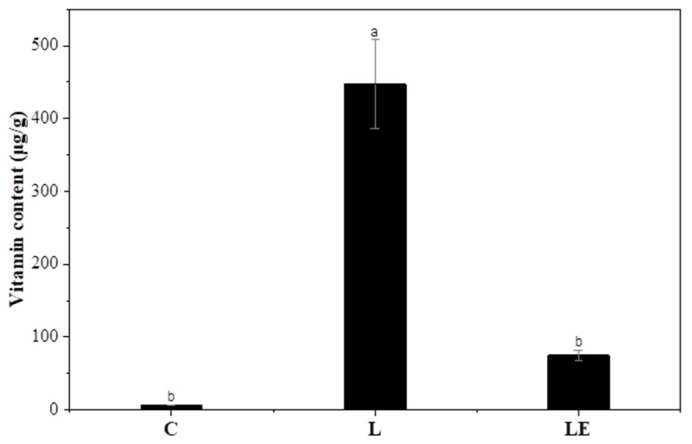
Vitamin content in garlic scapes treated with different liposome formulations. C, untreated sample; L, vitamin E-loaded liposome; LE, enzyme combined with vitamin E-loaded liposome. ^a,b^ Values with different superscript letters are significantly different (*p* < 0.05).

This section presents the vitamin E content of garlic scapes following treatment with L and LE, as assessed via HPLC (Figure 5). Compared to the control (C), both the L and LE treatments successfully delivered measurable amounts of vitamin E into the plant tissues, confirming the efficacy of liposomal delivery. This can be attributed to the fact that vitamin E is highly lipophilic and preferentially partitions into the phospholipid bilayer [18]. This observation aligns with previous studies demonstrating that liposomes enhance the solubility and bioavailability of lipophilic vitamins through their amphiphilic bilayer structures [48,49].

However, the L group showed a significantly higher vitamin E content than the LE group, suggesting that the presence of the enzyme may interfere with delivery efficiency. This could be owing to several factors, including altered liposome integrity upon enzyme binding, competition at the membrane interface, or reduced tissue uptake efficiency in the presence of both agents. Previous studies have shown that polysaccharides such as pectin and chitosan can stabilize liposomal membranes via hydrogen bonding and electrostatic interactions [50,51]. Consequently, enzymatic hydrolysis of these polysaccharides by pectinase may compromise interfacial stability and induce liposomal leakage, thereby lowering vitamin E retention. Similar studies have reported that liposomal enzymes can alter liposomal loading behavior, stability, and membrane integrity [52,53]. Nevertheless, the LE treatment exhibited the highest vitamin E content among the enzyme-treated groups, indicating that enzyme-assisted liposomal loading effectively enhanced the nutritional enrichment of garlic scapes. The enhanced vitamin E uptake in the LE group likely resulted from the synergistic effect of enzymatic permeabilization and liposomal delivery.

No detectable vitamin E was observed in C, B, E, or BE, indicating that vitamin E uptake was exclusively mediated by liposomal encapsulation. Therefore, the L treatment is optimal for achieving maximal vitamin E enrichment, and the LE treatment provides a dual advantage by promoting both enzymatic tenderization and vitamin E fortification. This combined effect highlights its potential as a promising approach for the development of functional food formulations targeting older adult consumers.

## 4. Conclusions

In this study, it was demonstrated that the application of dietary fiber-degrading enzymes, either alone or in combination with vitamin E-loaded liposomes, effectively modified the physicochemical and nutritional properties of garlic scapes. Enzyme treatment significantly influenced the color and texture profiles; specifically, Plantase UF, containing pectinase, β-glucanase and arabinose, provided a more controlled tenderization effect through partial loosening of the cellulose–hemicellulose matrix, whereas pectinase-rich Plantase PT caused extensive degradation of the pectic network in the middle lamella, resulting in excessive softening and higher adhesiveness. Microstructural analysis supported these observations, showing cell wall disruption and pore formation, with the Plantase PT-treated samples exhibiting more severe tissue collapse.

Liposome-based encapsulation influences enzyme performance and vitamin E delivery. Although co-loading of enzymes and vitamin E (LE) increased the particle size and reduced the ζ-potential, leading to decreased colloidal stability, encapsulation provided a protective effect that sustained enzyme activity during application. Vitamin E was successfully absorbed into garlic scape tissues, although vitamin E enrichment was more effective when vitamin E liposomes were used alone (L) than with the co-loaded formulations (LE).

In conclusion, enzyme–liposome systems can simultaneously achieve the tenderization and nutritional fortification of fibrous plant tissues. Such dual-function strategies hold potential for the development of older adult-friendly diets and functional vegetable-based products, although optimizing enzyme–nutrient interactions is essential for maximizing both the textural and nutritional benefits.

## Figures and Tables

**Table 1:** Color analysis of garlic scapes treated with different Plantase types, concentrations, and treatment durations.

Treatment	Concentration (%)	L*	a*	b*	*Δ*E
UF	0	35.08 ± 2.09 ^d^	−12.05 ± 0.84 ^b^	15.50 ± 1.40 ^bc^	-
	1	36.44 ± 2.15 ^bcd^	−10.68 ± 0.46 ^a^	15.10 ± 1.14 ^c^	3.75 ± 2.13 ^b^
	2	35.42 ± 0.57 ^cd^	−12.16 ± 0.49 ^b^	15.43 ± 0.71 ^bc^	0.20 ± 0.11 ^b^
	3	40.95 ± 2.56 ^a^	−12.32 ± 1.03 ^b^	18.66 ± 1.56 ^a^	27.26 ± 18.91 ^a^
PT	0	35.08 ± 2.09 ^d^	−12.05 ± 0.84 ^b^	15.50 ± 1.40 ^bc^	-
	1	37.60 ± 0.75 ^bc^	−11.17 ± 0.50 ^a^	15.39 ± 0.62 ^bc^	3.58 ± 1.17 ^b^
	2	35.41 ± 0.52 ^cd^	−12.09 ± 0.17 ^b^	15.31 ± 0.23 ^bc^	0.12 ± 0.08 ^b^
	3	37.79 ± 1.43 ^b^	−12.11 ± 0.52 ^b^	16.67 ± 0.68 ^b^	5.74 ± 4.60 ^b^
**Treatment**	**Treatment duration (h)**				
Control	0	35.08 ± 2.09 ^a^	−12.05 ± 0.84 ^a^	15.50 ± 1.40 ^a^	-
	12	37.43 ± 2.21 ^a^	−12.01 ± 0.68 ^a^	15.93 ± 1.37 ^a^	3.86 ± 4.97 ^a^
	24	35.08 ± 2.09 ^a^	−12.05 ± 0.84 ^a^	15.50 ± 1.40 ^a^	2.05 ± 2.07 ^a^
	48	35.20 ± 0.96 ^a^	−11.80 ± 0.70 ^a^	15.22 ± 0.74 ^a^	0.71 ± 0.62 ^a^
UF	0	35.08 ± 2.09 ^c^	−12.05 ± 0.84 ^a^	15.50 ± 1.40 ^c^	-
	12	38.89 ± 1.78 ^a^	−12.97 ± 0.23 ^bc^	18.07 ± 0.81 ^a^	12.91 ± 9.27 ^a^
	24	35.52 ± 1.38 ^bc^	−12.34 ± 0.87 ^ab^	16.55 ± 1.22 ^bc^	2.28 ± 0.73 ^b^
	48	37.43 ± 0.60 ^ab^	−13.47 ± 0.28 ^c^	17.62 ± 0.60 ^ab^	4.94 ± 0.99 ^ab^
PT	0	35.08 ± 2.09 ^ab^	−12.05 ± 0.84 ^ab^	15.50 ± 1.40 ^b^	-
	12	34.62 ± 2.17 ^b^	−11.71 ± 1.11 ^a^	15.78 ± 1.65 ^ab^	3.30 ± 2.79 ^a^
	24	37.41 ± 1.94 ^a^	−13.15 ± 0.76 ^b^	17.62 ± 1.42 ^a^	8.73 ± 8.41 ^a^
	48	35.37 ± 1.96 ^ab^	−12.29 ± 0.67 ^ab^	16.51 ± 1.63 ^ab^	4.05 ± 3.33 ^a^

Control, untreated enzyme; UF, Plantase UF; PT, Plantase PT. ^a–d^ Significant differences within the same treatment (enzyme concentration) and among treatments (treatment duration) (*p* < 0.05).

**Table 2:** Texture profile analysis of garlic scapes treated with different Plantase types and treatment durations.

**Treatment**	**Concentration (%)**	**Hardness (g)**	**Adhesiveness (mJ)**
UF	0	6046 ± 1088 ^ab^	0.54 ± 0.19 ^d^
	1	7236 ± 213 ^a^	0.38 ± 0.07 ^d^
	2	6861 ± 1323 ^a^	5.58 ± 1.94 ^b^
	3	6060 ± 666 ^ab^	0.48 ± 0.07 ^d^
PT	0	6046 ± 1088 ^ab^	0.54 ± 0.19 ^d^
	1	5132 ± 1525 ^bc^	4.58 ± 5.19 ^c^
	2	4101 ± 860 ^cd^	4.39 ± 2.21 ^bc^
	3	2815 ± 1062 ^d^	8.20 ± 2.01 ^a^
**Treatment**	**Treatment duration (h)**	**Hardness (g)**	**Adhesiveness (mJ)**
Control	0	6046 ± 1088 ^b^	0.54 ± 0.19 ^c^
	12	7518 ± 354 ^ab^	0.30 ± 0.09 ^c^
	24	7025 ± 1290 ^ab^	4.12 ± 0.39 ^bc^
	48	8781 ± 1241 ^a^	22.32 ± 2.82 ^a^
UF	0	6046 ± 1088 ^b^	0.54 ± 0.19 ^c^
	12	7053 ± 1842 ^ab^	0.20 ± 0.00 ^c^
	24	6861 ± 1323 ^ab^	5.58 ± 1.94 ^bc^
	48	7260 ± 1436 ^ab^	2.19 ± 0.30 ^bc^
PT	0	6360 ± 1187 ^b^	0.54 ± 0.19 ^c^
	12	5396 ± 438 ^b^	0.30 ± 0.11 ^c^
	24	5527 ± 495 ^b^	8.48 ± 5.58 ^b^
	48	2999 ± 1623 ^c^	28.34 ± 11.19 ^a^

Control, untreated enzyme; UF, Plantase UF; PT, Plantase PT. ^a–d^ Significant differences within the same treatment (enzyme concentration) and among treatments (treatment duration) (*p* < 0.05).

**Table 3:** Characterization of liposomes as affected by vitamin E concentration and enzyme loading.

Treatment	Particle Size (nm)	PdI	[-] ζ-Potential (mV)
V0	151 ± 2 ^e^	0.23 ± 0.01 ^c^	36.00 ± 1.73 ^a^
V0.025	1196 ± 71 ^b^	0.46 ± 0.09 ^b^	12.10 ± 0.30 ^cd^
V0.05	1306 ± 104 ^a^	0.32 ± 0.05 ^c^	11.43 ± 0.59 ^d^
V0.1	291 ± 12 ^d^	0.66 ± 0.04 ^a^	28.13 ± 1.23 ^b^
V0.2	404 ± 9 ^c^	0.65 ± 0.02 ^a^	13.73 ± 0.12 ^c^
E	575 ± 41 ^b^	0.79 ± 0.04 ^a^	13.57 ± 0.34 ^b^
L	291 ± 12 ^c^	0.66 ± 0.04 ^a^	28.13 ± 1.23 ^a^
LE	1214 ± 95 ^a^	0.81 ± 0.16 ^a^	12.97 ± 0.39 ^b^

V, vitamin E content in liposomes; E, 2% enzyme solution; L, vitamin E-loaded liposomes; LE, enzyme combined with vitamin E-loaded liposomes. ^a–e^ Significant differences within the same treatment (vitamin E concentration) and among treatments (liposome samples) (*p* < 0.05).

**Table 4:** Color analysis of garlic scapes treated with different liposome formulations.

Treatment	Treatment Duration (h)	L*	a*	b*	*Δ*E
C	3	35.33 ± 1.95 ^aBC^	−9.98 ± 1.04 ^aA^	12.76 ± 1.38 ^aC^	
	6	36.04 ± 1.21 ^aB^	−10.90 ± 0.28 ^aA^	13.77 ± 0.46 ^aD^	
	12	36.58 ± 3.12 ^aA^	−10.74 ± 0.78 ^aAB^	13.90 ± 1.41 ^aAB^	
E	3	37.02 ± 1.47 ^aAB^	−11.30 ± 0.72 ^aB^	14.89 ± 1.11 ^bB^	114.77 ± 20.54 ^aB^
	6	36.70 ± 0.78 ^aB^	−12.88 ± 1.20 ^bC^	17.21 ± 1.36 ^bB^	9.75 ± 5.66 ^bB^
	12	32.33 ± 2.14 ^bB^	−10.74 ± 1.14 ^aAB^	13.91 ± 1.74 ^aAB^	12.85 ± 9.21 ^bA^
B	3	34.33 ± 2.24 ^abC^	−10.42 ± 0.81 ^aA^	12.94 ± 0.90 ^bC^	87.17 ± 8.54 ^aC^
	6	36.13 ± 1.19 ^aB^	−12.75 ± 0.43 ^bC^	16.64 ± 0.48 ^aB^	6.62 ± 2.94 ^bB^
	12	11.49 ± 1.33 ^bB^	−10.16 ± 0.69 ^aA^	12.62 ± 1.24 ^bB^	10.99 ± 6.96 ^bA^
BE	3	34.62 ± 2.22 ^bC^	−10.40 ± 0.98 ^aA^	14.31 ± 1.18 ^bB^	105.89 ± 15.47 ^aB^
	6	41.77 ± 2.94 ^aA^	−14.18 ± 0.78 ^cD^	20.35 ± 1.40 ^aA^	48.26 ± 29.36 ^bA^
	12	33.90 ± 1.36 ^bB^	−11.44 ± 0.70 ^bB^	15.34 ± 0.94 ^bA^	6.22 ± 3.03 ^cA^
L	3	37.90 ± 1.78 ^aA^	−12.10 ± 0.34 ^cB^	16.80 ± 0.92 ^aA^	148.36 ± 20.43 ^aA^
	6	35.77 ± 0.32 ^bB^	−11.74 ± 0.13 ^bB^	15.56 ± 0.22 ^bC^	2.07 ± 0.47 ^bB^
	12	32.15 ± 0.60 ^cB^	−10.08 ± 0.31 ^aA^	12.46 ± 0.31 ^cB^	11.29 ± 3.01 ^bA^
LE	3	37.11 ± 0.51 ^aAB^	−11.89 ± 0.22 ^bB^	16.10 ± 0.35 ^aA^	133.22 ± 6.41 ^aA^
	6	32.54 ± 0.09 ^bC^	−10.31 ± 0.41 ^aA^	12.28 ± 0.44 ^bE^	7.53 ± 0.89 ^bB^
	12	33.61 ± 2.59 ^bB^	−11.68 ± 1.30 ^bB^	15.06 ± 2.24 ^aA^	11.11 ± 7.36 ^bA^

C, untreated sample; E, 2% enzyme solution; B, blank liposome; BE, enzyme combined with blank liposome; L, vitamin E-loaded liposome; LE, enzyme combined with vitamin E-loaded liposomes. ^a–c^ indicate significant differences within the same treatment across different treatment durations (*p* < 0.05). ^A–E^ indicate significant differences within the same treatment duration across different treatments (*p* < 0.05).

**Table 5:** Texture profile analysis of garlic scapes treated with different liposome formulations.

Treatment	Treated Duration (h)	Hardness (g)	Adhesiveness (mJ)
C	3	8697 ± 2010 ^aA^	3.24 ± 0.56 ^aA^
	6	7878 ± 2420 ^aAB^	2.30 ± 0.43 ^bB^
	12	9807 ± 1183 ^aA^	2.38 ± 0.83 ^bA^
B	3	8213 ± 921 ^abA^	3.18 ± 0.30 ^bA^
	6	7109 ± 828 ^bBC^	2.34 ± 0.26 ^bB^
	12	9144 ± 1626 ^aA^	2.37 ± 0.54 ^aA^
L	3	6708 ± 731 ^aAB^	3.32 ± 0.52 ^aA^
	6	9368 ± 518 ^aA^	1.80 ± 0.14 ^bC^
	12	8819 ± 2105 ^aAB^	2.75 ± 0.93 ^aA^
E	3	6862 ± 1652 ^abAB^	3.18 ± 0.13 ^aA^
	6	6618 ± 1550 ^bBC^	2.83 ± 0.44 ^aA^
	12	8882 ± 1622 ^aAB^	3.23 ± 0.40 ^aA^
BE	3	7236 ± 316 ^aAB^	3.28 ± 0.15 ^aA^
	6	5690 ± 781 ^aC^	2.86 ± 0.26 ^aA^
	12	6740 ± 1601 ^aC^	2.95 ± 0.50 ^aA^
LE	3	6098 ± 1369 ^aB^	3.33 ± 1.27 ^aA^
	6	6323 ± 874 ^aBC^	2.96 ± 0.44 ^aA^
	12	6816 ± 1579 ^aBC^	2.64 ± 0.70 ^aA^

C, untreated sample; B, blank liposome; L, vitamin E-loaded liposome; E, 2% enzyme solution; BE, enzyme combined with blank liposome; LE, enzyme combined with vitamin E-loaded liposomes. ^a–b^ indicate significant differences within the same treatment across different treatment durations (*p* < 0.05). ^A–C^ indicate significant differences within the same treatment duration across different treatments (*p* < 0.05).

**Table 6:** Particle size, polydispersity index (PdI), and ζ-potential of the remaining solution after treatment.

Treatment	Treatment Duration (h)	Particle Size (nm)	PdI	[-] ζ-Potential
B	3	157.30 ± 0.61 ^aD^	0.20 ± 0.01 ^bB^	35.57 ± 0.84 ^aA^
	6	160.20 ± 4.01 ^aB^	0.31 ± 0.05 ^aB^	36.63 ± 0.45 ^abA^
	12	156.43 ± 2.42 ^aC^	0.32 ± 0.03 ^aA^	34.87 ± 0.55 ^bB^
L	3	180.10 ± 3.60 ^bC^	0.32 ± 0.00 ^bA^	33.00 ± 2.21 ^bB^
	6	195.77 ± 9.07 ^aB^	0.39 ± 0.01 ^aA^	38.17 ± 4.19 ^ab^
	12	175.10 ± 1.80 ^bB^	0.32 ± 0.03 ^bA^	40.50 ± 3.55 ^aA^
BE	3	280.47 ± 2.79 ^aA^	0.21 ± 0.04 ^aB^	11.60 ± 0.46 ^bC^
	6	214.90 ± 3.95 ^bA^	0.13 ± 0.01 ^bD^	0.00 ± 0.81 ^cC^
	12	206.43 ± 2.50 ^cA^	0.19 ± 0.01 ^aB^	36.63 ± 0.82 ^aB^
LE	3	248.97 ± 8.17 ^aB^	0.25 ± 0.05 ^aB^	9.63 ± 0.50 ^aC^
	6	256.37 ± 5.80 ^aA^	0.20 ± 0.02 ^aC^	10.55 ± 1.33 ^bA^
	12	207.40 ± 3.24 ^bA^	0.13 ± 0.01 ^bC^	10.57 ± 0.32 ^aC^

B, blank liposomes; L, vitamin E-loaded liposomes; BE, enzyme combined with blank liposome; LE, enzyme combined with vitamin E-loaded liposomes. ^a–c^ indicate significant differences within the same treatment across different treatment durations (*p* < 0.05). ^A–D^ indicate significant differences within the same treatment duration across different treatments (*p* < 0.05).

## Data Availability

The original contributions presented in this study are included in the article/Supplementary Materials, further inquiries can be directed to the corresponding author.

## Supplementary Materials

The following supporting information can be downloaded at https://www.mdpi.com/article/10.3390/foods15010008/s1, Figure S1: Schematic diagram of the compression test for garlic scape samples.

## Abbreviations

The following abbreviations are used in this manuscript:

B	Blank liposome
BE	Enzyme combined with blank liposome
C	Untreated control sample
E	Enzyme solution
*Δ*E	Total color difference
L	Vitamin E-loaded liposome
LE	Enzyme combined with vitamin E-loaded liposome
PdI	Polydispersity index
PT	Plantase PT
UF	Plantase UF
V	Vitamin E content in liposomes
