# Supplementary figures and images for: Enzyme-Assisted Tenderization and Vitamin E-Loaded Liposome Coating for Garlic Scape Quality Enhancement
Source: Foods. 2025 Dec 19;15(1):8. doi: 10.3390/foods15010008 (PMC12786241; doi:10.3390/foods15010008)

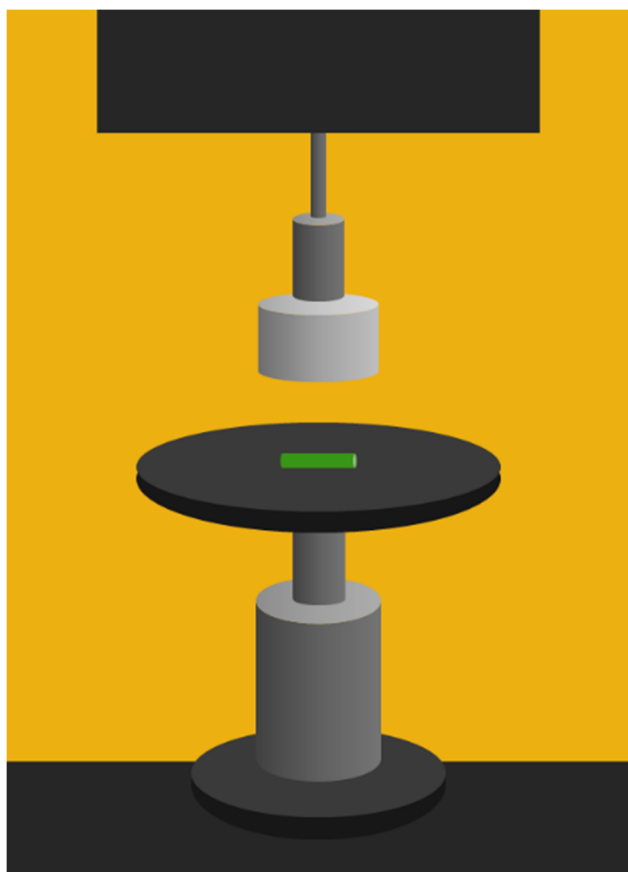

**Figure S1.** Schematic diagram of the compression test for garlic scape samples.

Supplement: Supplementary file 1 [file foods-15-00008-s001.zip › foods-4028861-supplementary.pdf]
